# Clinical course of congestive hepatopathy pre/post heart transplantation

**DOI:** 10.1007/s00508-023-02231-2

**Published:** 2023-06-28

**Authors:** Lorenz Balcar, Georg Semmler, Bernhard Scheiner, Albert Friedrich Stättermayer, Stefan Ćosić, Philipp Schwabl, Niema Kazem, Mattias Mandorfer, Martin Hülsmann, Andreas Zuckermann, Thomas Reiberger

**Affiliations:** 1 Division of Gastroenterology and Hepatology, Department of Internal Medicine III, Medical University of Vienna, Waehringer Guertel 18–20, 1090 Vienna, Austria; 2 Vienna Hepatic Hemodynamic Lab, Division of Gastroenterology and Hepatology, Department of Internal Medicine III, Medical University of Vienna, Vienna, Austria; 3 Christian-Doppler Laboratory for Portal Hypertension and Liver Fibrosis, Medical University of Vienna, Vienna, Austria; 4 Department of Cardiology, Medical University of Vienna, Vienna, Austria; 5 Department of Cardiac Surgery, Medical University of Vienna, Vienna, Austria

**Keywords:** HTX, Cirrhosis, Cardiac cirrhosis, Congestive hepatopathy, Ischemic hepatitis

## Abstract

**Background and aims:**

Heart failure (HF) might lead to increased hepatic venous pressure, thereby impairing hepatic blood outflow and subsequently inducing congestive hepatopathy. We aimed to evaluate prevalence of congestive hepatopathy in patients undergoing heart transplantation (HTX) as well as their post-transplant course.

**Methods:**

Patients undergoing HTX from 2015–2020 at the Vienna General Hospital were included (*n* = 205). Congestive hepatopathy was defined by hepatic congestion on abdominal imaging and hepatic injury. Laboratory parameters, ascites severity, and clinical events were assessed and post-HTX outcomes evaluated.

**Results:**

At listing, 104 (54%) patients showed hepatic congestion, 97 (47%) hepatic injury, and 50 (26%) had ascites. Congestive hepatopathy was diagnosed in 60 (29%) patients, who showed more often ascites, lower serum sodium and cholinesterase activity, and higher hepatic injury markers. Mean albumin-bilirubin (ALBI)-score as well as (modified)-model for end-stage liver disease (MELD)-scores were higher in patients with congestive hepatopathy. Median levels of laboratory parameters/scores normalised after HTX, and ascites resolved in most patients with congestive hepatopathy (*n* = 48/56, 86%). The post-HTX (median follow-up 55.1 months) survival was 87% and liver-related events were rare (3%). Severe ascites, low cholinesterase, and MELD/MELD-XI were associated with ascites persistence/death 1‑year after HTX. Age, male sex, and severe ascites were the only independent predictors of post-HTX mortality. Both ALBI and MELD-scores were robust indicators of post-HTX survival when measured 4 weeks after HTX (ALBI log-rank test *p* < 0.001; MELD log-rank test *p* = 0.012).

**Conclusion:**

Congestive hepatopathy and ascites were mostly reversible after HTX. Liver-related scores and ascites improve prognostication in patients after HTX.

## Introduction

Congestive liver disease represents a type of liver pathology which is caused by impaired blood outflow from the liver [1]. This pathology is mostly caused by heart failure (HF) [2]. Chronic hepatic congestion is a term used to describe long lasting blood flow congestion in the liver veins. Liver fibrosis may occur around the hepatic veins in a process described as congestive induration [2]. Small nodules of regenerating liver tissue can be observed (focal nodular hyperplasia [FNH]-like lesions) which may lead to false diagnoses of cirrhosis, usually designated as ‘cardiac cirrhosis’ [3]. In prolonged congestive disease the increase in pressure of hepatic veins leads to creation of hepatic vein-to-vein shunts [3]. The exact mechanism as to why this happens is still unknown and the current theory suggests the preferential retrograde flow from the inferior vena cava into the right hepatic vein to be the cause [3].

While many patients are asymptomatic or show only unspecific symptoms, some patients may experience dull right upper quadrant pain secondary to stretching of the liver capsule [4]. In most cases the course of the disease is mild, and the patients display no significant symptoms [4]. Laboratory parameters may vary [1, 4]. Serum aminotransferases are increased in about one third of patients, whereas increased serum levels of bilirubin and alkaline phosphatase have been observed in 20% of cases [5]. Radiomorphologically, dilatation of hepatic veins (5–6 millimetres in healthy subjects), flow changes, change in diameter and congestion in the inferior vena cava are mostly observed [3].

Treatment of congestive hepatopathy relies mostly on restoral of heart function which will remove hepatic venous congestion. Furthermore, the prevalence and course of congestive hepatopathy in patients undergoing heart transplantation (HTX) remains largely unknown. Therefore, our aim was to evaluate (i) the prevalence of congestive hepatopathy in patients undergoing HTX as well as (ii) its clinical course, (iii) the natural history of ascites in these patients, and (iv) factors associated with post-HTX survival.

## Methods

### Study design and population

In this single-centre, retrospective study, patients listed for HTX at the Department of Cardiac Surgery of the General Hospital Vienna (AKH), Austria between 01/2015 and 12/2020 were included. We only included adult (≥ 18 years) patients. Patients in whom data was insufficient were excluded from this study. Demographic, clinical, and relevant laboratory parameters were collected at listing, prior to HTX, 4 weeks post HTX, 1 year post HTX and at the last follow-up from available medical records.

### Clinical, laboratory, and imaging parameters

The parameters collected within this study included parameters of hepatic injury and hepatic function, blood counts, virus serology, and metabolic parameters/comorbidities/comedications. Radiologic data was evaluated prior to as well as approximately 1 year after HTX. Ascites was graded as none, mild/moderate and severe. The 2016-updated United Network for Organ Sharing Model for end-stage liver disease (MELD) was calculated. It was calculated by incorporating sodium into the original MELD score (MELD[i] = 0.957 × ln[Cr] + 0.378 × ln[bilirubin] + 1.120 × ln[INR] + 0.643; MELD = MELD[i] + 1.32 × [137-Na] − (0.033 × MELD[i] × [137-Na])) [6]. Next to the MELD score, the MELD-excluding INR [7] (MELD-XI = 5.11 × ln[bilirubin] + 11.76 × ln[creatinine] + 9.44) [7] as well as the modified MELD score (Albumin-MELD) [8], replacing INR with albumin levels to substitute impaired production of coagulation factors (as reflected by INR) with albumin (albumin > 41 g/dL: Albumin-MELD[i] = 0.957 × ln[Cr] + 0.378 × ln[bilirubin] + 1.120 × ln[1] + 0.643; albumin ≤ 41 g/dL: Albumin-MELD[i] = 0.957 × ln[Cr] + 0.378 × ln[bilirubin] + 1.120 × ln[41-albumin] + 0.643; Albumin-MELD = Albumin-MELD[i] + 1.32 × [137-Na] − (0.033 × MELD[i] × [137-Na])) were calculated [8]. In addition, the Albumin-Bilirubin (ALBI) score was calculated (ALBI score = −0.085 × [albumin] + 0.66 × ln[bilirubin]) [9], and graded as ALBI-Grade‑1 (≤ −2.6, preserved hepatic function), ALBI-Grade‑2 (> −2.6 to ≤ −1.39, mild hepatic dysfunction), and ALBI-Grade‑3 (> −1.39, severe hepatic dysfunction). To assess the severity of heart insufficiency, N‑terminal pro b‑type natriuretic peptide (NT-proBNP) levels were recorded.

### Criteria and definition of congestive hepatopathy

Congestive hepatopathy was defined as a combination of hepatic venous congestion on abdominal imaging and hepatic injury indicated by elevated levels of hepatic transaminase or gamma-glutamyl transferase levels. Evidence of hepatic venous congestion on abdominal imaging was defined by enlarged hepatic veins or reverse flow in hepatic veins. Evidence of hepatic injury was defined by an elevation of alanine aminotransferase > 1.5 of the upper limit of normal (women > 42 U/L, men > 75 U/L), and/or the elevation of gamma-glutamyl transferase > 1.5 of the upper limit of normal (women > 38 U/L, men > 60 U/L).

### Statistical analysis

Statistical analyses were performed using IBM SPSS Statistics 27 (IBM, New York, NY, USA), or GraphPad Prism 8 (GraphPad Software, CA, USA). Categorical variables were reported as absolute (*n*) and relative frequencies (%), whereas continuous variables as mean ± standard deviation (SD) or median (interquartile range [IQR]), as appropriate. Student’s t‑test was used for group comparisons of normally distributed variables and Mann-Whitney-U-test for non-normally distributed variables. Group comparisons of categorical variables were performed using either Pearson’s Chi-squared or Fisher’s exact test. Median follow-up time was calculated using the reverse Kaplan-Meier method from date of inclusion to death/last follow-up date. Next, variables which were significantly different among baseline characteristics, significantly associated with the outcome of interest in univariable analysis were included into a multivariable Cox regression model as covariables. Primary endpoint was post-HTX survival, with presence of ascites/death 1 year after HTX as secondary combined endpoint. Survival analyses were demonstrated by Kaplan-Meier curves and compared by means of the log-rank test. To evaluate parameters associated with ascites/death at 1 year post HTX, logistic regression analyses were performed. Sankey plots were used for graphical representation of different courses of disease during follow-up. A *p*-value of < 0.05 was considered statistically significant.

## Results

### Patient characteristics

**Fig. 1:**
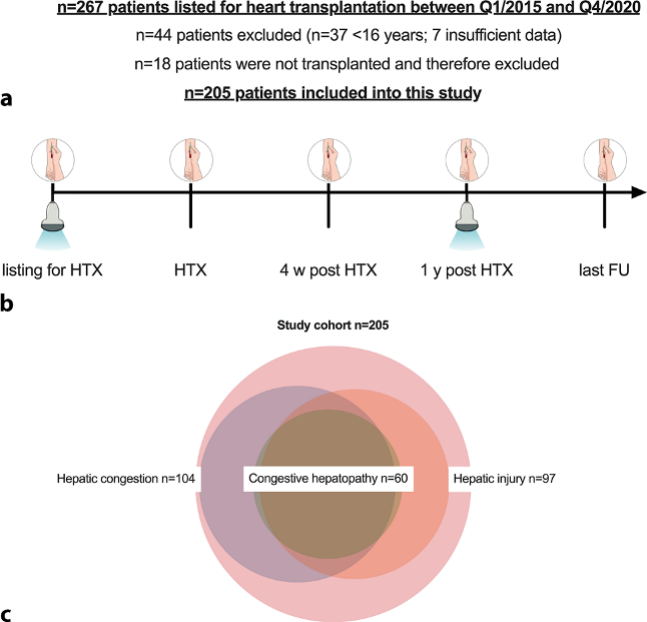
Patient flowchart (**a**), (**b**) study design and (**c**) Venn-diagram demonstrating the prevalence of hepatic congestion/injury and congestive hepatopathy at study inclusion (Abbreviations: *HTX* heart transplantation; *FU* follow-up; *w* weeks; *y* years)

Overall, 267 patients were evaluated for inclusion. After applying in- and exclusion criteria, 205 patients could finally be included into this study (Fig. 1). While data on baseline imaging was available in 193 patients (94%), follow-up imaging was available in 175 (85%).

Mean age of the overall cohort was 54.0 ± 13.5 years (*n* = 147, 72% males) and mean BMI was 25.4 ± 4.0 kg/m^2^. Dilatative (*n* = 103, 50%) and ischemic (*n* = 87, 42%) cardiomyopathies were the main aetiologies of heart failure, while other/unclassified cardiomyopathies (*n* = 15, 8%) were rare. Almost every fourth patient had diabetes at study inclusion (*n* = 50, 24%). Significant alcohol consumption was rare (*n* = 8, 4%). Comedications are summarised in Table 1.

**Table 1:** Patient characteristics at the time of HTX listing

Variables	All patients *n* = 205 (100%)	Cong. Hep.^a^ *n* = 60 (29%)	No Cong. Hep.^a^ *n* = 133 (65%)	*p*-value
**Age, mean** **±** **SD, years**	54.0 ± 13.5	54.5 ± 13.8	53.6 ± 13.8	0.676
**Male sex, *n* (%)**	147 (72)	39 (65)	98 (74)	0.219
**BMI, mean** **±** **SD, kg/m**^**2**^	25.4 ± 4.0	24.5 ± 3.5	25.7 ± 3.9	*0.041*
**Etiology of heart failure, *n* (%)**				
Dilatative cardiomyopathy	103 (50)	35 (58)	62 (47)	*0.008*
Ischemic cardiomyopathy	87 (42)	17 (28)	65 (49)	
Other cardiomyopathy	15 (8)	8 (14)	6 (4)	
**Diabetes, *n* (%)**	50 (24)	12 (20)	37 (28)	0.248
**VAD prior to surgery, *n* (%)**	54 (26)	7 (12)	47 (35)	*<* *0.001*
**Renal failure, *n* (%)**	36 (18)	12 (20)	21 (16)	0.472
**Reported alcohol consumption, *n* (%)**				
None	150 (73)	36 (60)	106 (80)	*0.010*
Moderate	47 (23)	19 (32)	24 (18)	
Significant	8 (4)	5 (8)	3 (2)	
**Medications, *n* (%)**				
Betablockers	144 (70)	40 (67)	100 (75)	0.220
ACE inhibitors	87 (42)	26 (43)	58 (44)	0.971
ARB	38 (19)	10 (17)	27 (20)	0.553
MRA	136 (66)	39 (65)	91 (68)	0.639
Diuretics	166 (81)	46 (78)	112 (84)	0.208
Sacubitril/valsartane	18 (9)	4 (7)	13 (10)	0.481
Digoxin	9 (4)	3 (5)	5 (4)	0.706
Anti-platelet therapy	134 (65)	33 (55)	96 (72)	*0.019*
Anticoagulation	163 (80)	49 (82)	104 (78)	0.582
Statins	84 (41)	17 (28)	64 (48)	*0.010*
Amiodarone	44 (22)	14 (23)	30 (23)	0.905
**Radiological findings, *n* (%)**^a^				
*Ascites*				
None	143 (74)	36 (60)	107 (81)	*0.010*
Mild/moderate (grade 1–2)	39 (20)	18 (30)	21 (16)	
Severe (grade 3)	11 (6)	6 (10)	5 (4)	
*Liver steatosis*	19 (10)	7 (12)	12 (9)	0.580
*Splenomegaly*	45 (22)	16 (27)	29 (22)	0.460
*Hepatic congestion*	104 (54)	60 (100)	44 (33)	*<* *0.001*
**Laboratory parameters, mean** **±** **SD or median (IQR)**				
Hemoglobin, g/dL	12.2 ± 2.3	12.2 ± 2.6	12.1 ± 2.3	0.815
Platelet count, G/L	220 ± 83	220 ± 90	221 ± 81	0.941
INR	1.7 ± 0.8	1.8 ± 0.9	1.7 ± 0.8	0.412
Sodium, mmol/L	137.7 ± 4.5	136.8 ± 4.7	138.2 ± 4.4	*0.037*
Creatinine, mg/dL	1.2 (1.0–1.8)	1.4 (1.0–2.0)	1.2 (1.0–1.6)	0.074
Bilirubin, mg/dL	0.8 (0.5–1.4)	1.2 (0.7–1.6)	0.7 (0.4–1.1)	*<* *0.001*
Albumin, g/dL	40.1 ± 7.4	39.1 ± 7.6	40.5 ± 7.4	0.230
Alkaline phosphatase, U/L	86 (65–118)	113 (91–151)	42 (36–46)	*<* *0.001*
Aspartate aminotransferase, U/L	27 (22–39)	30 (24–47)	25 (20–36)	*0.002*
Alanine aminotransferase, U/L	26 (17–38)	28 (18–49)	24 (16–35)	0.101
Gamma-glutamyl transferase, U/L	75 (39–138)	159 (118–265)	49 (32–96)	*<* *0.001*
NT-proBNP, pg/mL	3374 (1627–7827)	5253 (2115–9891)	2815 (1237–7113)	*0.014*
Cholinesterase, U/L	5.7 ± 2.3	4.7 ± 1.8	6.1 ± 2.3	*<* *0.001*
Lactate dehydrogenase, U/L	235 (190–299)	245 (198–309)	229 (183–286)	0.165
**Clinical scores, mean** **±** **SD, points**				
MELD	17.2 ± 6.7	18.9 ± 7.9	16.4 ± 6.1	*0.033*
MELD-XI	14.3 ± 5.1	15.6 ± 5.5	13.6 ± 4.8	*0.006*
Albumin-MELD	19.9 ± 11.4	23.2 ± 11.5	18.6 ± 11.3	*0.009*
ALBI score	−2.7 ± 0.7	−2.5 ± 0.7	−2.7 ± 0.7	*0.024*
*ALBI stages, n (%)*				
1	124 (61)	28 (47)	88 (66)	*0.025*
2	68 (33)	28 (47)	36 (27)	
3	13 (6)	4 (7)	9 (7)	
**Virology, *n* (%)**				
HBs antigen/HBV-DNA pos	1 (0.5)/0	1 (2)/0	–	0.311
HCV antibody/HCV-RNA pos	2 (1)/0	–	2 (2)/0	1.000
CMV IgG/CMV-DNA pos	126 (62)/0	36 (60)/0	85 (64)/0	0.603

*ACE* angiotensin converting enzyme; *ALBI score* albumin-bilirubin score; *Albumin-MELD* modified Model for End-Stage Liver Disease replacing INR by Albumin-to-upper-normal-limit ratio; *ARB* angiotensin receptor blockers; *BMI* body mass index; *CMV* cytomegaly virus; *HBs* hepatitis B virus surface; *HCV* hepatitis C virus; *INR* international normalised ratio; *IQR* interquartile range; *MELD* Model for End-Stage Liver Disease; *MELD-XI* Model for End-Stage Liver Disease excluding INR; *MRA* mineralocorticoid receptor antagonists; *n* number; *NT-proBNP* N-terminal pro b‑type natriuretic peptide; *SD* standard deviation; *VAD* ventricular assist device

^a^Available in 193 patients (94%)

Radiological findings at baseline reveal that one fourth of patients had ascites (*n* = 39, 20% mild/moderate; *n* = 11, 6% severe). Liver steatosis was not very common (*n* = 19, 10%) whereas 22% of patients had splenomegaly (*n* = 45). Hepatic venous congestion was diagnosed in 104 patients (54%) at imaging, while hepatic injury was present in 97 (47%). Baseline laboratory parameters are provided in Table 1. Mean MELD was 17.2 ± 6.7, while mean MELD-XI was 14.3 ± 5.1, mean Albumin-MELD was 19.9 ± 11.4, and mean ALBI score was −2.7 ± 0.7 points (*n* = 124, 61% ALBI grade 1; *n* = 68, 33% ALBI grade 2; *n* = 13, 6% ALBI grade 3).

Congestive hepatopathy was diagnosed in 60 patients (29%). When comparing patients with vs. without congestive hepatopathy, patients with congestive hepatopathy had significantly lower BMI values (24.5 ± 3.5 vs. 25.7 ± 3.9 kg/m^2^; *p* = 0.041). Patients with congestive hepatopathy had more often dilatative and other cardiomyopathies as underlying diseases for HF. Reported alcohol consumption was higher in patients with congestive hepatopathy compared to patients without. Moreover, they had more often ascites (40% vs. 20%; *p* = 0.010). Interestingly, use of anti-platelet therapy, statins, calcium channel blockers and levothyroxine substitution were significantly different. Patients with congestive hepatopathy also had lower serum sodium levels (136.8 ± 4.7 vs. 138.2 ± 4.4 mmol/L; *p* = 0.037), higher bilirubin, alkaline phosphatase, aspartate aminotransferase, gamma-glutamyl transferase (according to its definition), as well as lower cholinesterase (4.7 ± 1.9 vs. 6.1 ± 2.4 U/L; *p* < 0.001) but higher C‑reactive protein (1.2 [IQR: 0.5–2.8] vs. 0.4 [IQR: 0.2–1.8] mg/dL; *p* = 0.004) and NT-proBNP values (5253 [IQR: 2115–9891] vs. 2815 [IQR: 1237–7113] pg/mL; *p* = 0.014). Considering clinical scores, patients with congestive hepatopathy had significantly higher MELD score (18.9 ± 7.9 vs. 16.4 ± 6.1 points; *p* = 0.031), MELD-XI (15.6 ± 5.5 vs. 13.6 ± 4.8 points; *p* = 0.006), Albumin-MELD (23.2 ± 11.5 vs. 18.6 ± 11.3 points; *p* = 0.009) and ALBI score values (−2.5 ± 0.7 vs. −2.7 ± 0.7 points; *p* = 0.021).

### Comparison of evolution of laboratory parameters pre/post HTX among patients with vs. without congestive hepatopathy

**Fig. 2:**
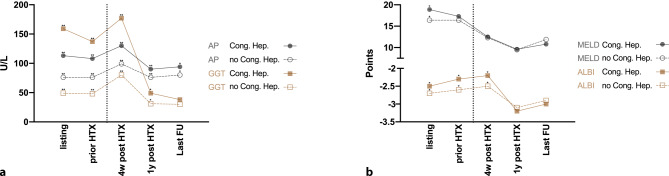
Course of laboratory parameters reflecting (**a**) hepatic injury (i.e., ALP and GGT) and (**b**) hepatic function (MELD and ALBI score) at different study time points and comparison between patients with vs. without congestive hepatopathy (Abbreviations: *ALBI* albumin-bilirubin score; *AP* alkaline phosphatase; *GGT* gamma-glutamyl transferase; *HTX* heart transplantation; *FU* follow-up; *MELD* Model for End-Stage Liver Disease) (*Denotes *p* < 0.05; ** denotes *p* < 0.001)

Table 2 demonstrates measured laboratory parameters across different study time points. Most importantly, alkaline phosphatase as marker for hepatic injury was significantly higher prior to and 4 weeks/1 year after HTX when comparing patients with vs. without congestive hepatopathy. Accordingly, gamma-glutamyl transferase levels were also significantly higher; however, both injury markers decreased after HTX and were below the upper limit of normal at last follow-up (Fig. 2a). This could be demonstrated for each laboratory parameter included in our study, as depicted in Table 2. Furthermore, clinical scores reflecting hepatic function (i.e., MELD and ALBI/MELD-XI and Albumin-MELD scores) were comparable after HTX (Fig. 2b).

**Table 2:** Comparison of the evolution of different parameters at different study time points between patients with vs. without congestive hepatopathy

Variables mean ± SD or median (IQR)	Cong. Hep. *n* = 60 (29%)					No Cong. Hep. *n* = 133 (65%)				
	At listing *n* = 60	Prior HTX *n* = 60	4w post HTX *n* = 59	1y post HTX *n* = 51	Last FU *n* = 60	At listing *n* = 133	Prior HTX *n* = 133	4w post HTX *n* = 129	1y post HTX *n* = 120	Last FU *n* = 133
Hemoglobin, g/dL	12.2 ± 2.6	12.1 ± 2.3	10.1 ± 1.4*	12.6 ± 1.9	13.0 ± 2.3	12.1 ± 2.3	12.2 ± 2.1	10.8 ± 2.7*	12.6 ± 1.7	12.8 ± 2.5
Platelet count, G/L	220 ± 90	208 ± 99	299 ± 90*	233 ± 66	223 ± 81	220 ± 81	208 ± 78	260 ± 88*	231 ± 58	206 ± 79
White blood count, G/L	7.9 ± 3.2	8.6 ± 3.0	7.8 ± 3.4	5.5 ± 1.6	6.6 ± 3.5	8.4 ± 3.8	8.8 ± 4.0	7.9 ± 3.3	6.1 ± 2.0	7.4 ± 4.5
INR	1.8 ± 0.9	1.5 ± 0.6	1.1 ± 0.3	1.0 ± 0.1	1.1 ± 0.3	1.7 ± 0.8	1.6 ± 0.8	1.0 ± 0.1	1.0 ± 0.1	1.2 ± 0.4
Sodium, mmol/L	136.8 ± 4.7*	135.9 ± 5.8*	138.0 ± 4.2	140.6 ± 2.6	139.4 ± 3.0	138.2 ± 4.4*	137.6 ± 4.8*	137.5 ± 3.7	140.3 ± 2.7	139.9 ± 3.4
Creatinine, mg/dL	1.4 (1.0–2.0)	1.2 (1.1–1.7)	1.3 (1.0–1.7)	1.2 (1.0–1.5)	1.1 (0.9–1.5)	1.2 (1.0–1.6)	1.2 (1.0–1.6)	1.2 (1.0–1.6)	1.1 (0.9–1.4)	1.1 (0.9–1.5)
Bilirubin, mg/dL	1.2 (0.7–1.6)**	1.2 (0.6–1.8)*	0.6 (0.4–0.7)	0.5 (0.4–0.8)	0.6 (0.4–0.8)	0.7 (0.4–1.1)**	0.7 (0.4–1.4)*	0.5 (0.3–0.7)	0.5 (0.4–0.7)	0.6 (0.4–1.0)
Albumin, g/dL	39.1 ± 7.6	37.2 ± 7.4	33.5 ± 7.2*	45.1 ± 5.1*	43.7 ± 6.8	40.6 ± 7.5	39.4 ± 7.9	36.4 ± 6.1*	43.6 ± 3.6*	42.8 ± 7.9
Alkaline phosphatase, U/L	113 (91–151)**	108 (74–158)**	130 (94–182)**	90 (71–132)*	94 (69–116)	76 (57–100)**	76 (50–104)**	99 (78–133)**	76 (59–93)*	80 (66–105)
Aspartate aminotransferase, U/L	30 (24–47)*	31 (22–56)	25 (17–33)*	28 (19–33)*	23 (19–31)	25 (20–36)*	27 (21–41)	19 (16–26)*	23 (19–29)*	24 (19–32)
Alanine aminotransferase, U/L	28 (18–49)	24 (15–38)	23 (16–36)	25 (18–37)*	22 (16–34)	23 (16–35)	23 (17–36)	21 (15–29)	21 (15–27)*	21 (15–35)
Gamma-glutamyltransferase, U/L	159 (118–265)**	137 (80–248)**	177 (108–303)**	49 (32–149)**	38 (21–140)*	49 (32–93)**	48 (29–90)**	80 (46–147)**	31 (19–49)**	30 (18–69)*
NT-proBNP, pg/mL	5253 (2115–9891)*	4757 (2329–8230)	2189 (978–4860)	333 (145–522)	368 (128–785)	2815 (1237–7113)*	2787 (1052–5843)*	2728 (1427–5954)	368 (202–721)	400 (184–1043)
Cholinesterase, mean ± SD, U/L	4.7 ± 1.9**	4.6 ± 1.7**	4.4 ± 1.8	7.3 ± 1.4	7.5 ± 2.0	6.1 ± 2.4**	6.2 ± 2.4**	4.5 ± 1.4	6.8 ± 1.7	6.9 ± 2.2
Lactate dehydrogenase, U/L	245 (198–309)	250 (195–309)	247 (188–303)	204 (173–253)	189 (153–219)	228 (183–286)	230 (187–309)	231 (189–276)	208 (172–257)	189 (165–228)
C‑reactive protein, mg/dL	1.2 (0.5–2.8)*	1.2 (0.6–2.7)**	0.9 (0.4–3.3)	0.2 (0.1–0.4)	0.2 (0.1–0.4)	0.4 (0.2–1.8)*	0.5 (0.2–1.6)**	0.6 (0.3–1.8)	0.1 (0.1–0.4)	0.2 (0.1–1.0)
*Clinical scores, points*										
MELD	18.9 ± 7.9*	17.3 ± 6.2	12.5 ± 5.8	9.6 ± 2.9	10.8 ± 4.7	16.4 ± 6.1*	16.4 ± 5.7	12.3 ± 5.2	9.5 ± 2.8	11.9 ± 5.9
MELD-XI	15.6 ± 5.5*	15.3 ± 5.1	13.8 ± 5.4	12.4 ± 2.9	12.5 ± 3.9	13.6 ± 4.8*	14.0 ± 4.6	13.2 ± 4.2	11.9 ± 3.1	13.4 ± 5.4
Albumin-MELD	23.2 ± 11.5*	25.1 ± 11.1*	28.2 ± 11.9*	10.2 ± 4.8	13.4 ± 9.6	18.6 ± 11.3*	19.4 ± 12.3*	24.2 ± 11.4*	10.4 ± 6.2	14.4 ± 11.4
ALBI score	−2.5 ± 0.7*	−2.3 ± 0.7*	−2.2 ± 0.6*	−3.2 ± 0.4	−3.0 ± 0.6	−2.7 ± 0.7*	−2.6 ± 0.8*	−2.5 ± 0.6*	−3.1 ± 0.3	−2.9 ± 0.8

*ALBI score* albumin-bilirubin score; *Albumin-MELD* Albumin-modified Model for End-Stage Liver Disease replacing INR by Albumin-to-ULN ratio; *HTX* heart transplantation; *INR* international normalised ratio; *IQR* interquartile range; *FU* follow-up; *MELD* Model for End-Stage Liver Disease; *MELD-XI* Model for End-Stage Liver Disease excluding INR; *n* number; *SD* standard deviation

*Denotes *p*-values < 0.05

**Denotes *p*-values < 0.001

### Natural history of ascites in patients with vs. without congestive hepatopathy

**Fig. 3:**
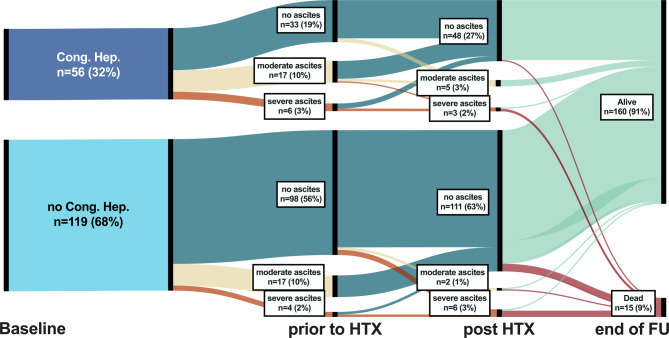
Sankey plot depicting the course of ascites in patients with vs. without congestive hepatopathy for the study time points (i) prior HTX, (ii) 1 year after HTX, and (iii) at the end of follow-up (Abbreviations: *FU* follow-up; *HTX* heart transplantation)

Figure 3 demonstrates that not only laboratory markers and hepatic injury/function indicators were normalising after HTX, but also ascites. Interestingly, while ascites was more common in patients with congestive hepatopathy at baseline, ascites regression was comparable in patients with vs. without congestive hepatopathy post-HTX. However, persistence of ascites remained an indicator for poor post-HTX survival, unrelated to congestive hepatopathy status prior to HTX.

### Clinical course of patients with vs. without congestive hepatopathy

Median time on HTX list was 1.7 months and median post-HTX survival was 41.8 months. Five patients had a large-volume paracentesis during follow-up (2%), while 2 of them also had a spontaneous bacterial peritonitis (1%). Variceal bleeding, hepatic encephalopathy as well as hepatocellular carcinoma were not observed. Overall, 26 patients died within the study period (13%).

While MELD and Albumin-MELD scores were associated with post-HTX survival in univariable Cox regression analysis (MELD: hazard ratio [HR]: 1.06 [95% confidence interval [CI]: 1.00–1.12]; *p* = 0.040; Albumin-MELD: 1.04 [95%CI: 1.00–1.07]; *p* = 0.029), age, male sex, and severe ascites remained independently associated with post-HTX survival in multivariable analysis (Table 3). This could be demonstrated for different multivariable models. To evaluate factors associated with presence of ascites/death 1 year after HTX, logistic regressions were calculated, demonstrating that severe ascites (HR: 7.20 [95%CI: 1.94–26.68]; *p* = 0.003), cholinesterase (HR: 0.75 [95%CI: 0.61–0.92]; *p* = 0.007) as well as MELD (HR: 1.09 [95%CI: 1.02–1.16]; *p* = 0.007) and MELD-XI scores (HR: 1.10 [95%CI: 1.02–1.18]; *p* = 0.012) were associated with the combined secondary endpoint of interest. In multivariable analysis, no factor remained independently associated with ascites/death at 1 year after HTX (Table 4). However, MELD/MELD-XI/ALBI scores tended to be associated with the outcome of interest as well as severe ascites.

**Table 3:** Cox regression models with variables associated with post-HTX mortality

Variables	Univariable		Multivariable Model 1 (including MELD score)		Multivariable Model 2 (including modMELD score)		Multivariable Model 3 (including ALBI score)	
	HR (95%CI)	*p*-value	aHR (95%CI)	*p*-value	aHR (95%CI)	*p*-value	aHR (95%CI)	*p*-value
*Age, per year*	1.04 (1.00–1.08)	0.053	1.05 (1.00–1.10)	*0.033*	1.05 (1.01–1.10)	*0.028*	1.05 (1.00–1.10)	*0.031*
*Male sex (vs. female)*	3.25 (0.98–10.83)	0.055	5.00 (1.15–21.78)	*0.032*	4.99 (1.15–21.56)	*0.031*	5.62 (1.29–24.38)	*0.021*
*BMI, per kg/m*^*2*^	0.99 (0.95–1.04)	0.778	–	–	–	–	–	–
*VAD prior to surgery*	1.59 (0.71–3.56)	0.263	–	–	–	–	–	–
*Etiology of heart failure*								
Dilatative cardiomyopathy (reference)	1	**–**	–	–	–	–	–	–
Ischemic cardiomyopathy	0.99 (0.44–2.21)	0.981	–	–	–	–	–	–
Other cardiomyopathy	1.25 (0.28–5.54)	0.770	–	–	–	–	–	–
*Months on transplant list, per month*	0.96 (0.90–1.02)	0.201	–	–	–	–	–	–
*Reported alcohol consumption*								
None (reference)	1	**–**	–	–	–	–	–	–
Moderate/Significant	0.85 (0.34–2.12)	0.727	–	–	–	–	–	–
*Ascites grade at baseline*								
None (reference)	1	**–**	1	–	1	–	1	–
Mild/Moderate	1.72 (0.70–4.22)	0.237	1.99 (0.75–5.33)	0.170	2.11 (0.83–5.38)	0.118	2.23 (0.88–5.66)	0.093
Severe	3.19 (0.92–11.03)	0.067	4.69 (1.04–21.14)	*0.044*	4.85 (1.10–21.34)	*0.037*	5.08 (1.14–22.66)	*0.033*
*Congestive hepatopathy (vs. absent)*	0.54 (0.20–1.44)	0.218	–	–	–	–	–	–
*Creatinine, per mg/dL*	1.09 (0.84–1.40)	0.518	–	–	–	–	–	–
*Albumin, per g/dL*	0.97 (0.92–1.02)	0.245	–	–	–	–	–	–
*Cholinesterase, per U/L*	0.85 (0.70–1.02)	0.083	0.96 (0.79–1.16)	0.679	1.02 (0.81–1.28)	0.889	0.92 (0.71–1.18)	0.490
*C‑reactive protein, per mg/dL*	0.99 (0.90–1.08)	0.808	–	–	–	–	–	–
*MELD, per point*	1.06 (1.00–1.12)	*0.040*	1.02 (0.96–1.09)	0.495	–	–	–	–
*MELD-XI, per point*	1.06 (0.99–1.14)	0.122	–	–	–	–	–	–
*Albumin-MELD, per point*	1.04 (1.00–1.07)	*0.029*	–	–	1.02 (0.98–1.07)	0.379	–	–
*ALBI score, per point*	1.35 (0.78–2.32)	0.280	–	–	–	–	0.81 (0.36–1.80)	0.597

*ALBI* Albumin-Bilirubin score; *Albumin-MELD* Albumin-modified Model for End-Stage Liver Disease replacing INR by Albumin-to-ULN ratio; *HTX* heart transplantation; *MELD* Model for End-Stage Liver Disease; *MELD-XI* Model for End-Stage Liver Disease excluding INR; *VAD* ventricular assist device

**Table 4:** Logistic regression model with variables associated with the compositive outcome of death and new onset/persisting ascites within 1 year after HTX

Variables	Univariable		Multivariable Model 1 (including MELD score)		Multivariable Model 2 (including MELD-XI score)		Multivariable Model 3 (including ALBI score)	
	HR (95%CI)	*p*-value	aHR (95%CI)	*p*-value	aHR (95%CI)	*p*-value	aHR (95%CI)	*p*-value
*Age, per year*	1.00 (0.97–1.03)	0.804	–	–	–	–	–	–
*Male sex (vs. female)*	1.88 (0.67–5.26)	0.229	–	–	–	–	–	–
*BMI, per kg/m*^*2*^	0.91 (0.81–1.02)	0.118	–	–	–	–	–	–
*VAD prior to surgery*	1.33 (0.55–3.18)	0.527	–	–	–	–	–	–
*Etiology of heart failure*								
Dilatative cardiomyopathy (reference)	1	**–**	–	–	–	–	–	–
Ischemic cardiomyopathy	0.50 (0.20–1.22)	0.128	–	–	–	–	–	–
Other cardiomyopathy	0.99 (0.20–5.02)	0.994	–	–	–	–	–	–
*Months on transplant list, per month*	0.98 (0.93–1.03)	0.437	–	–	–	–	–	–
*Reported alcohol consumption*								
None (reference)	1	**–**	–	–	–	–	–	–
Moderate/Significant	0.90 (0.36–2.28)	0.822	–	–	–	–	–	–
*Ascites grade at baseline*								
None (reference)	1	**–**	1	–	1	–	1	–
Mild/Moderate	2.31 (0.89–6.00)	0.087	1.53 (0.54–4.33)	0.428	1.65 (0.59–4.59)	0.337	1.91 (0.70–5.24)	0.208
Severe	7.20 (1.94–26.68)	*0.003*	3.45 (0.76–15.56)	0.108	3.56 (0.79–16.04)	0.098	3.88 (0.87–17.22)	0.075
*Congestive hepatopathy (vs. absent)*	1.34 (0.57–3.13)	0.505	–	–	–	–	–	–
*Creatinine, per mg/dL*	1.19 (0.91–1.55)	0.210	–	–	–	–	–	–
*Albumin, per g/dL*	0.98 (0.93–1.03)	0.462	–	–	–	–	–	–
*Cholinesterase, per U/L*	0.75 (0.61–0.92)	*0.007*	0.85 (0.68–1.07)	0.174	0.85 (0.68–1.07)	0.174	0.72 (0.53–0.98)	*0.034*
*C‑reactive protein, per mg/dL*	1.00 (0.91–1.09)	0.949	–	–	–	–	–	–
*MELD, per point*	1.09 (1.02–1.16)	*0.007*	1.06 (0.99–1.13)	0.072	–	–	–	–
*MELD-XI, per point*	1.10 (1.02–1.18)	*0.012*	–	–	1.06 (0.98–1.15)	0.130	–	–
*Albumin-MELD, per point*	1.03 (0.99–1.07)	0.101	–	–	–	–	–	–
*ALBI score, per point*	1.24 (0.69–2.21)	0.470	–	–	–	–	0.52 (0.22–1.20)	0.125

*ALBI* Albumin-Bilirubin score; *Albumin-MELD* Albumin-modified Model for End-Stage Liver Disease replacing INR by Albumin-to-ULN ratio; *HTX* heart transplantation; *MELD* Model for End-Stage Liver Disease; *MELD-XI* Model for End-Stage Liver Disease excluding INR; *VAD* ventricular assist device

**Fig. 4:**
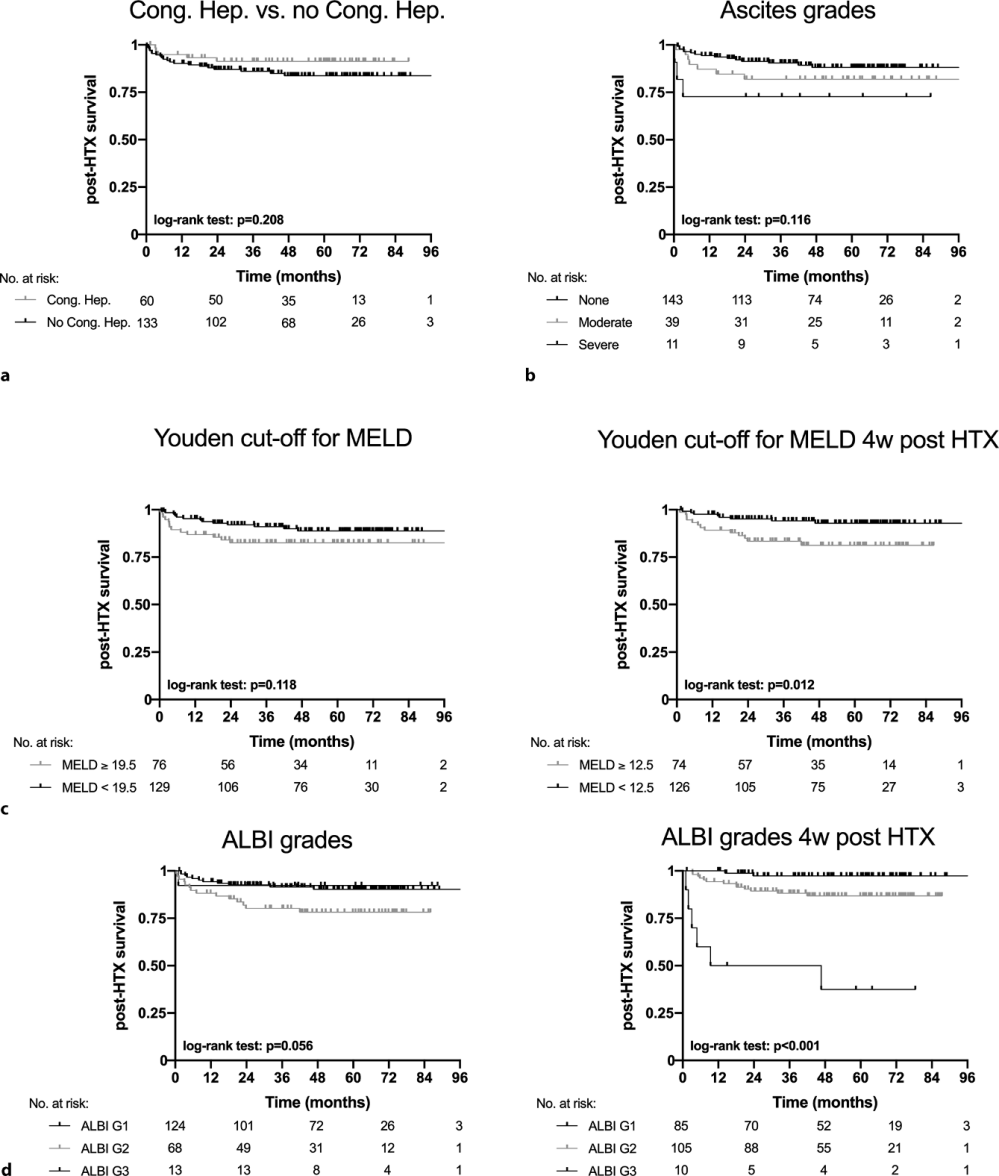
Kaplan-Meier curves demonstrating post-heart transplantation (*HTX*) survival according to (**a**) congestive hepatopathy at HTX, (**b**) ascites graduation at HTX, (**c**) MELD score cut-offs as well as (**d**) ALBI-score grades at HTX and at 4 weeks after HTX (Abbreviations: *ALBI score* albumin-bilirubin score; *HTX* heart transplantation; *MELD* Model for End-Stage Liver Disease)

Figure 4a shows that diagnosis of congestive hepatopathy at HTX did not influence post-HTX survival (log-rank test *p* = 0.208). Ascites graduation tended to differentiate into distinct clinical courses (log-rank test *p* = 0.116; Fig. 4b). When stratifying patients according to hepatic function at HTX, graduation with ALBI score (log-rank test *p* = 0.056) could identify patients at high risk. Both ALBI and MELD scores were robust indicators of post-HTX survival when measured 4 weeks after HTX (ALBI log-rank test *p* < 0.001; MELD log-rank test *p* = 0.012; Fig. 4c, d).

## Discussion

This is the first study demonstrating the role of congestive hepatopathy and ascites in patients undergoing HTX in a large, well-characterized cohort. We could identify patients with congestion/hepatic injury at HTX listing, characterised them descriptively and showed that by treating the underlying disease (i.e., HTX), regression of congestive hepatopathy is common. In line, ascites regression was observed in most of included patients independent of congestive hepatopathy status prior to HTX. Furthermore, we could identify and confirm potential markers for risk prediction in this specific cohort of patients.

Potential reversibility of liver function tests 3 months post HTX were already demonstrated by Dichtl et al. [10]. In line, Chokshi et al. confirmed these results also for a long-term follow-up period and underlined the prognostic impact of the MELD score [8]. Moreover, to overcome the potential bias of anticoagulants deriving the INR, they modified the MELD score by replacing INR by albumin [8]. Importantly, we confirm these results [8, 10]. We also included and confirmed the prognostic value of the MELD-XI score. However, next to the MELD also the ALBI score has prognostic implications in post-HTX survival. Ascites might improve risk stratification in patients with congestive hepatopathy.

Overall, neither congestive hepatopathy, nor liver dysfunction or congestive hepatopathy prior to HTX were associated with post-HTX survival. Liver-related events were very rare (3%) and post-HTX survival was good (85%, median follow-up 55 months). Ascites prior to HTX tended to be a negative prognostic indicator (Fig. 4b). However, ascites mostly resolved after HTX. In patients in whom ascites remained, proposed clinical scores should be used for guidance of further management of congestive hepatopathy. It must be mentioned that 20% of patients without congestive hepatopathy also presented with ascites but without hepatic injury. 27% of these patients had also renal failure and we can only speculate about other reasons for ascites formation (e.g., malnutrition). While around two thirds of patients with hepatic injury also presented with hepatic congestion on imaging, one third did not. In those patients, hepatic injury might be caused by low cardiac output/left ventricular forward failure.

Other natural history studies/studies on the clinical course of congestive hepatopathy have been performed more than 20 years ago [5, 11]. The changes in HF aetiology (from rheumatic valvular disease to ischemic cardiomyopathy) and in daily clinical practice as well as improvements in HTX surgery and availability of HTX warrant the need of contemporary studies targeting congestive hepatopathy. These improvements overall have already reduced the incidence of cardiac cirrhosis [12] (median time on waiting list was 1.7 months in our study) and underlined the need of studies targeting earlier stages of congestive hepatopathy.

Our study describing the natural course of patients presenting with congestive hepatopathy prior/post HTX has several strengths: First, due to the homogenous cohort and high rates of imaging results available, our study accurately represents the clinical course of patients with congestive hepatopathy prior/post HTX. Second, in contrast to most previous studies, we focused on congestion/hepatic injury in a selected cohort of patients listed for HTX. Our results demonstrate that graduation of ascites provides additional important information for clinical risk stratification. Moreover, the impact of hepatic function (i.e., especially MELD and ALBI scores) on the clinical outcomes is distinct for this study population. Third, the follow-up period with a median duration of 55 months (i.e., more than 4 years) enabled a detailed evaluation of the different clinical courses of congestive hepatopathy.

However, this study also has its limitations: To ensure the homogeneity of the cohort, patients who were not transplanted were excluded from this study. However, numbers were low (*n* = 18), and we are confident that our results are firm. Next, due to the retrospective design of the study, we cannot exclude that some liver-related or other events have been missed. However, we have thoroughly reviewed all individual electronic health records of the hospital associations and nation-wide electronic health records. Furthermore, baseline imaging was available in only 193 (94%) and follow-up imaging in 175 patients (85%). Accordingly, follow-up results on ascites regression/progression are missing in 15% of patients and hence, future prospective cohort studies should confirm our results for the clinical course of ascites in patients with congestive hepatopathy. Due to the retrospective design of the study, liver biopsies and liver vein catheterisation for hepatic haemodynamics were not performed in all patients in a study-specific standardised setting so that we can only demonstrate data on the clinical course of these patients. However, given the very benign course of congestive hepatopathy (13% of patients died), invasive procedures should only be performed to rule out cardiac cirrhosis and hepatic haemodynamics should only be performed in a study-related setting. Non-invasive tests to improve risk stratification is warranted and subject of future prospective trials.

In conclusion, congestive hepatopathy and ascites are mostly reversible after HTX. Liver-related scores are associated with post-HTX survival. Regarding prognostication, ascites improves risk stratification in patients with congestive hepatopathy undergoing HTX. Therefore, a detailed hepatic evaluation prior to HTX might be warranted in HF patients with ascites and high ALBI or MELD.

## Abbreviations

ALBI score: Albumin-Bilirubin score
Albumin-MELD: Modified Model for End-Stage Liver Disease replacing INR by Albumin-to-upper-normal-limit ratio
FNH: Focal nodular hyperplasia
(a) HR: (adjusted) Hazard ratio
HF: Heart failure
HTX: Heart transplantation
HVPG: Hepatic venous pressure gradient
INR: International normalised ratio
IQR: Interquartile range
MELD: Model for End-Stage Liver Disease
MELD-XI: Model for End-Stage Liver Disease excluding INR
*n*: Number
NT-proBNP: N‑terminal pro b‑type natriuretic peptide
PPG: Portal pressure gradient
SD: Standard deviation
UNOS MELD (2016): United Network for Organ Sharing Model for End-Stage Liver Disease (2016)
